# Conservation risks for paper collections induced by the microclimate in the repository of the Alessandrina Library in Rome (Italy)

**DOI:** 10.1186/s40494-022-00714-5

**Published:** 2022-06-10

**Authors:** Elena Verticchio, Francesca Frasca, Patrizia Cavalieri, Lorenzo Teodonio, Daniela Fugaro, Anna Maria Siani

**Affiliations:** 1 Department of Earth Sciences, Sapienza Università di Roma, P.le A. Moro 5, 00185 Rome, Italy; 2 Department of Physics, Sapienza Università di Roma, P.le A. Moro 5, 00185 Rome, Italy; 3 Istituto Centrale per la Patologia degli Archivi e del Libro, Via Milano 76, 00184 Rome, Italy; 4 Biblioteca Universitaria Alessandrina, P.le A. Moro 5, 00185 Rome, Italy

**Keywords:** Microclimate monitoring, Historic library repository, Paper collections, Risk assessment, Preventive conservation, Temperature, Relative humidity, Colorimetric measurements

## Abstract

The Alessandrina Library was founded in 1667 by pope Alexander VII Chigi and is nowadays housed in the Campus of Sapienza University of Rome (Italy). Within its Ancient (mostly made of rag paper) and Modern (mostly made of contemporary paper) collections, it includes more than one million books produced from the XVI to the XXI century. In 2019, six thermo-hygrometers were deployed in its multi-storey repository to monitor temperature (T) and relative humidity (RH). Hourly T and RH data collected over 2 years allowed us to evaluate spatial and temporal thermo-hygrometric distributions and to carry out a comprehensive assessment of the climate-induced risks (mechanical, chemical, and biological deterioration mechanisms). Vertical temperature gradients associated with unstable conditions occurred in winter, resulting in upraising air flows up to the ceiling. The risky short-term RH fluctuations (EN 15757:2010) were determined to avoid mechanical stress in case of loans, relocation, and consultation. The Time Weighted Expected Lifetime (TWEL) index was used to evaluate the chemical risk for different paper-based collections as a function of their acidity and degree of polymerisation, also considering the typical response time of paper books to T and RH changes. The TWEL calculation estimated that the durability of acidic paper was around 300 years and highlighted that rag paper could be subject to cellulose hydrolysis only in summer and autumn, while contemporary paper was mostly at no risk. The risk of mould germination (Sedlbauer diagram) was possible on few days in Autumn, while the production of insect eggs (Brimblecombe empirical function) was favoured during approximately 42% of time over the year. In addition, illuminance and colorimetric measurements (performed on selected book covers) showed that light-sensitive objects could be exposed to the photodeterioration risk in the east-facing side of the repository. Although the investigation focussed on a specific case study, a similar approach could be effectively adapted to most library and archival repositories conserving paper-based collections.

## Introduction

The durability of library collections can be threatened by deterioration processes driven by the environmental conditions. Hence, the management strategies for preserving historical libraries must entail the mitigation of climate-induced deterioration risks. Although digitisation of collections can reduce to a certain extent the risks due to handling [1], it is, however, pivotal to guarantee the durability of the materials constituting historic libraries, as they are non-renewable resources from our past. The assessment of the microclimate allows an objective evaluation of the climate-induced deterioration risks, with a view to planning tailored preventive conservation actions [2].

Library collections are made of a wide range of hygroscopic organic materials (e.g., paper, leather, parchment, cloth), which are climate-sensitive and, therefore, vulnerable to deterioration. Paper is usually the most widely occurring material in libraries in the Mediterranean region, as it has been extensively used since the XI-XII centuries [3]. Paper typically contains cellulose, a natural polymer forming long chains (i.e., fibres), and can be classified into three main types—rag, acidic, and contemporary—as a function of their different acidity (pH) and degree of polymerisation (DP). The average values of pH and DP for rag, acidic, and contemporary paper can be retrieved from the Collection Demography App platform [4], where pre-set values for the corresponding paper types were provided based on the SurveNIR reference collection [5]. Rag paper is a high-quality paper made from cotton (originally from cotton rags and nowadays from cotton linters) characterised, on average, by pH = 6.4 and DP = 1481.2. Acidic machine-made paper (produced from the XIX century due to the growing demand for printing media) is a poor-quality paper with short wood fibres and usually contains lignin and acidic chemicals, with an average pH = 5.2 and DP = 826.3. Contemporary paper is made from highly processed wood pulp with alkaline reserves, with average pH = 7.6 and DP = 1526.2. As the hygroscopic materials continuously exchange moisture with the air, the time needed for them to reach equilibrium with the environmental temperature and humidity changes (i.e., their response time) must be considered while performing the climate-induced risk assessment. In the case of paper materials, attention should be paid to moisture exchanges both in small boxes (e.g., in microclimate frames [6] or compact shelving system) and inside libraries (open shelving system) [7, 8].

The principal risks affecting the durability of library collections include mechanical, chemical, biological and photodeterioration mechanisms. Several recent studies, summarised in [9], have investigated the microclimate data collected in libraries considering various standards and guidelines for heritage conservation, as well as using different methods to assess the deterioration risks.

Handling is frequently responsible for the accumulation of wear and tear on paper [10], thus reducing the durability of the collections [11]. Moreover, since library materials can shrink/swell as they lose/gain moisture, temperature and relative humidity fluctuations can be studied as mechanical stressors potentially inducing dimensional changes in library objects [11, 12].

Cellulose hydrolysis is among the main climate-induced risks for paper collections. This mechanism is largely driven by temperature and is usually estimated by using dose–response functions expressing the chemical risk as a function of temperature and relative humidity [13–15]. The effect of pollutants on historic paper deterioration is generally negligible [16]. However, dust particles and particulate matter may increase paper vulnerability to deterioration [17, 18].

Fungal spores, which are ubiquitous, become a biological risk when RH > 65% for a prolonged time [19]. Air purity, although pollutants (e.g., NO and SO_2_) might depress mould growth, is not usually considered in spore germination and mycelial growth prediction models [20]. Insect development is directly related to temperature (i.e., insects rapidly proliferate in warm conditions) and relative humidity (e.g., eggs and young larvae can be sensitive to dehydration) [21].

Visible light, and particularly UV radiation (if not filtered out), can accelerate embrittlement of poor-quality paper and cause yellowing [22] and/or colour fading of most dyes, inks and colourants [23].

### Research aims

The aim of this work is to investigate the principal climate-induced deterioration risks for paper collections in the repository of the Alessandrina Library (Rome, Italy). To this end, the microclimate observations collected for 2 years were analysed. Section 2 deals with the site description, the microclimate monitoring campaign and the observations collected. The methods for characterising the indoor climate and assessing the conservation risks are also described here. Section 3 is devoted to presentation and discussion of the results. Section 4 outlines the main conclusions of the work.

## Materials and methods

### Case study: collection and building metadata

**Fig. 1:**
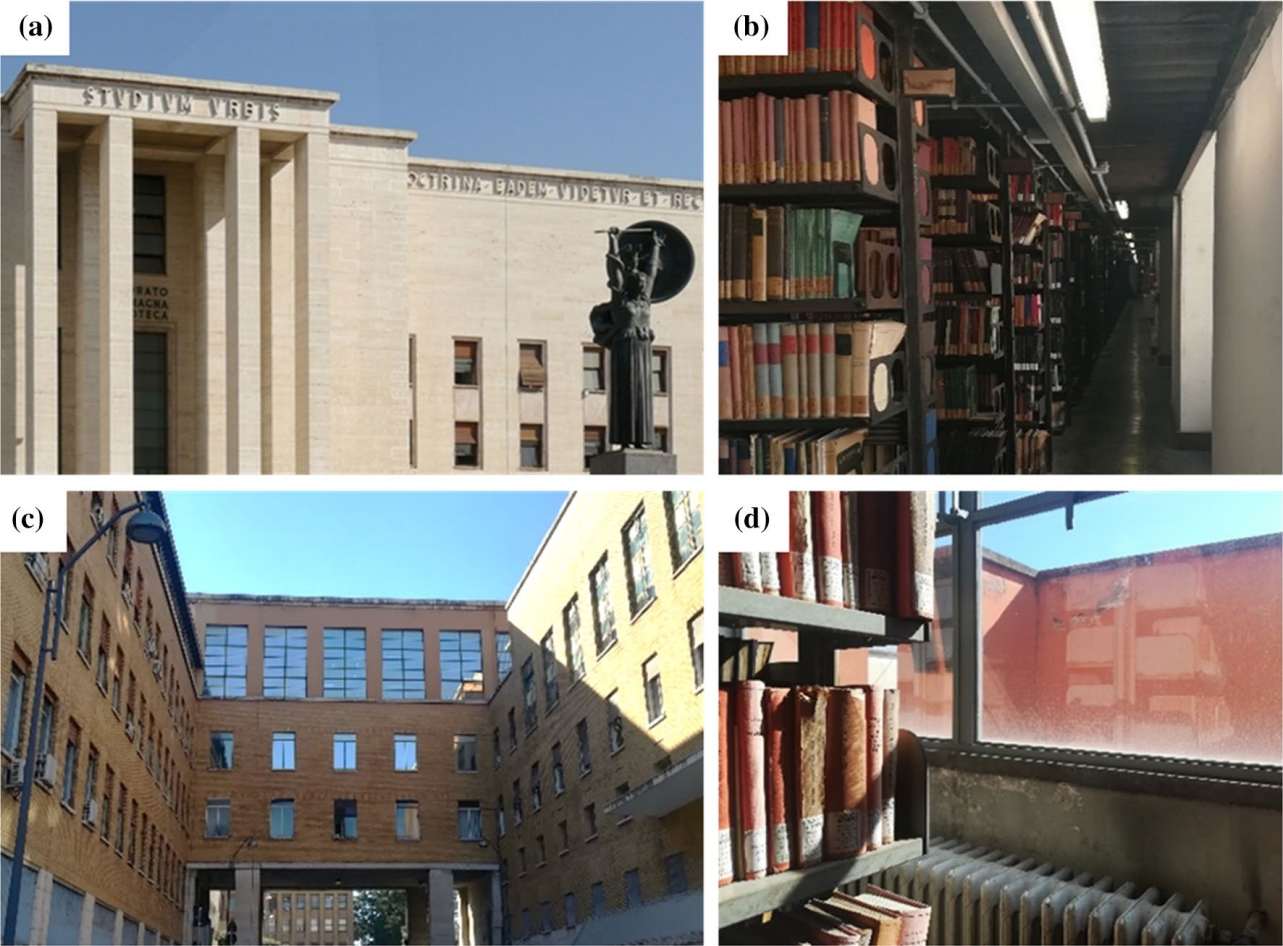
Front (**a**) and back (**c**) view of the Rectorate building of Sapienza University of Rome; west-oriented (**b**) and east-oriented (**d**) windows in the Alessandrina library repository

The Alessandrina Library is among the 46 prestigious libraries belonging to the Italian Ministry of Culture and preserves one of the most important university collections in Italy. The Library was founded in 1667 by Pope Alexander VII Chigi as the Library of the *Studium Urbis,* the University of Rome*.* Originally housed in the Roman Baroque church of Sant'Ivo alla Sapienza (designed by the architect Francesco Borromini from 1642 to 1660), its historical nucleus includes duplicates from the Chigiana Library, the Vatican Library and the valuable library of the Dukes of Urbino. From 1935, the Alessandrina Library was relocated in the upper floors (from third to fifth) of the Rectorate building (Fig. 1a) within the Campus of Sapienza University in Rome (Lat. 41.9° N and Long. 12.5° E, 21 m a.m.s.l.). The Library also includes the pre-existing libraries of the Faculties of Humanities, Law and Political Science, as well as important donations. Nowadays, Alessandrina was elected as the legal repository for all the documents of cultural interest destined to public use and published by editors from the province of Rome. High-resolution digital pictures and metadata of some of the most valuable documents in the Alessandrina collection are available on the web [24, 25].

**Fig. 2:**
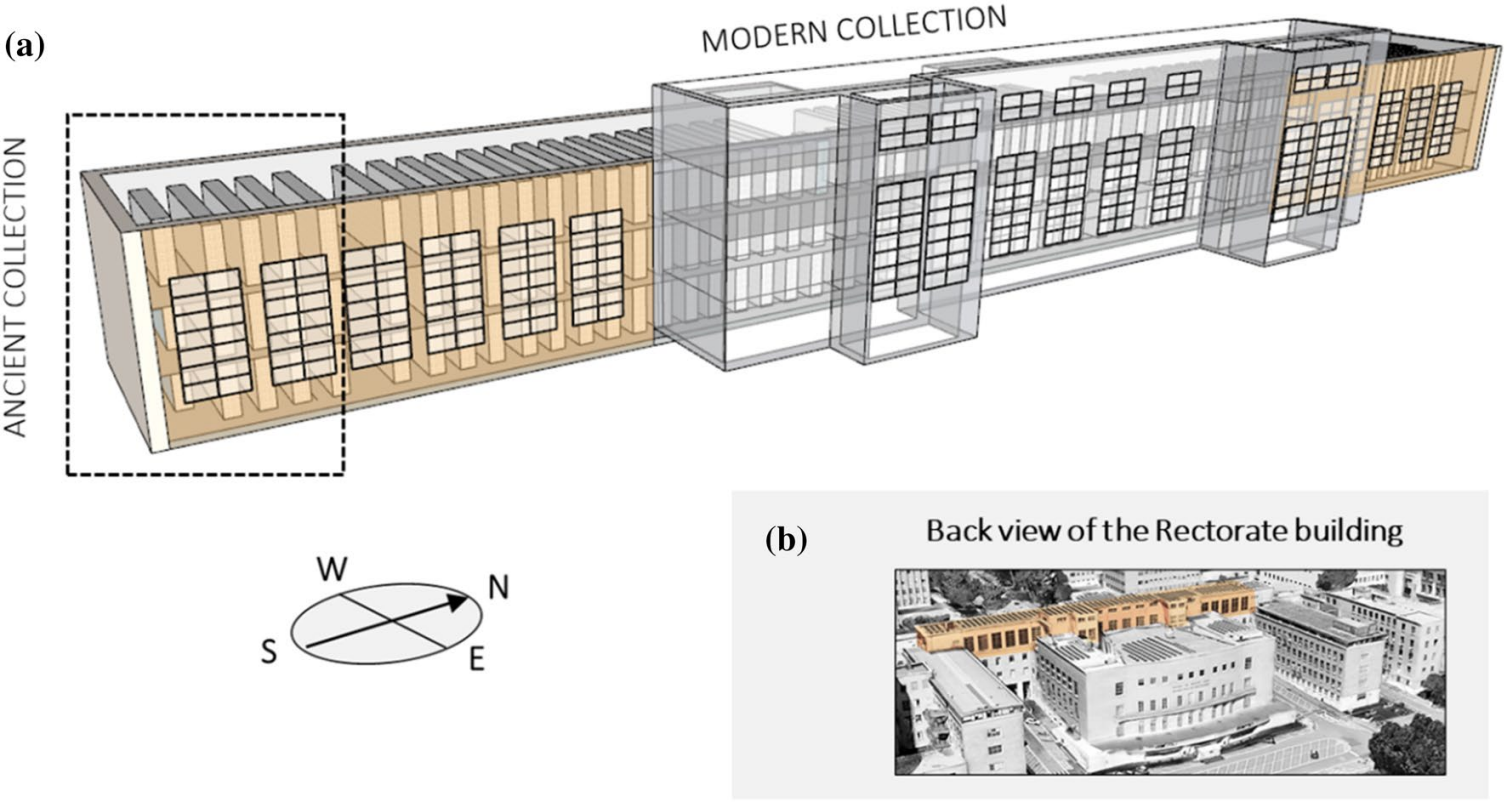
(**a**) Sketch of the Rectorate building portion where the Alessandrina library repository is housed, (**b**) rear aerial view of the building (the major repository block is highlighted in orange)

The Alessandrina repository, accessible only by the Library staff, is divided into two main blocks (Fig. 2a). The multi-storey repository has a total volume of more than 4000 m^3^, with a ceiling about 7 m high and a floor area wider than 500 m^2^. Windows are located on both the east and west walls of the repository and have single-pane glasses with UV-filters, which have severely deteriorated over time. The west-oriented windows (total area of approximately 30 m^2^) face the façade of the Rectorate building and are shaded under the Rectorate prostyle (a-b). These windows are frequently open during the warmer months. On the back of the Rectorate building, larger east-oriented windows (total area of approximately 85 m^2^) are exposed to direct natural light during the diurnal hours (Fig. 1c, d). These windows are mostly closed during the year. The repository is naturally ventilated and equipped with fan coils for both heating and cooling. However, the air conditioning system is obsolete and scarcely effective in controlling temperature over the year.

The Alessandrina collection includes two major assets based on the period of production of the books: the hereafter called Ancient collection, deployed on three floors in the south-facing part of the repository (Fig. 2a, dashed rectangle), and the hereafter called Modern collection, which occupies the rest of the repository space (Fig. 2). Books are densely packed onto open metal shelves. Paper is the prevalent material in both the Ancient and the Modern collection assets. The Ancient collection is mostly made of rag paper produced from XVI to XIX century with around 3% of parchment. The Modern collection is mostly made of XX-XXI century contemporary paper, with small amounts of cardboard, plastics and canvas.

### Monitoring campaign

The microclimate conditions were monitored only in the major block of the repository (Fig. 2a, highlighted in orange). The indoor climate was monitored for two full years, from 1st August 2019 until 31st July 2021, to study the hygrothermal behaviour of the repository and to carry out a comprehensive assessment of the climate-induced risks.

Two microclimate field campaigns were conducted before the installation of the sensors to identify representative sampling points to evaluate the spatial distribution of heat and moisture. The microclimate field measurements were carried out in spring 2019 during different times of the day (morning/afternoon) on the horizontal cross-sections of the repository on a 4 m × 4 m regular grid at 1.5 m above the floor using portable instruments manufactured by Rotronic (model HygroPalm). Microclimate contour maps were elaborated from T and MR observations and showed that the T distribution was affected by the solar radiation entering from the east-facing windows during the morning (which locally increased the temperatures up to + 2 °C), while the MR distribution was homogeneous along the horizontal section. This preliminary investigation was useful to identify representative sampling points for deploying the sensors.

**Fig. 3:**
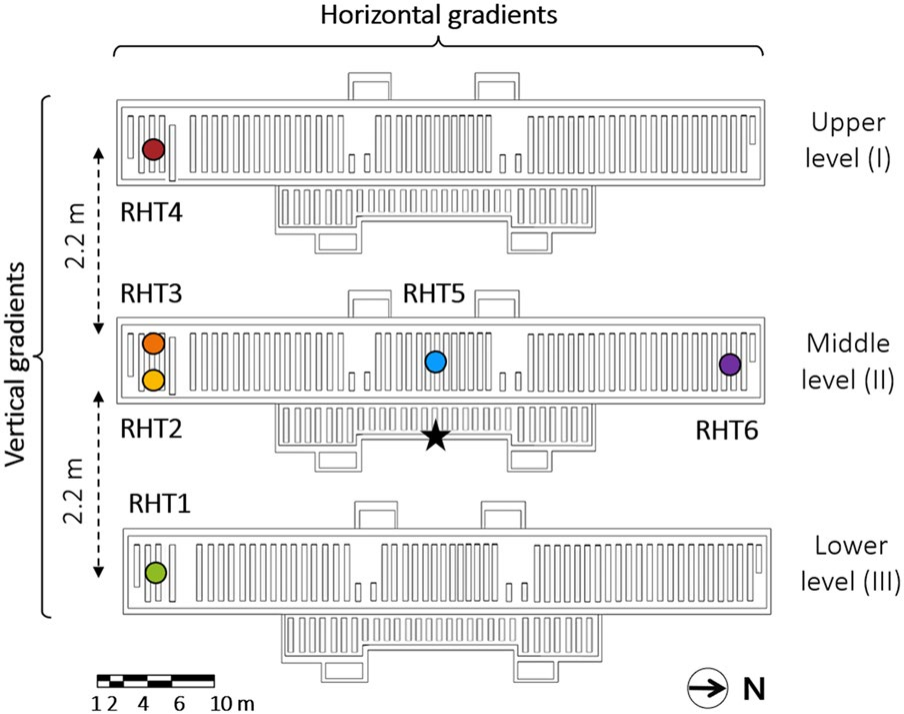
Position of the six thermo-hygrometers (RHT), indicated as coloured markers, and of the light sensor, indicated as a black star. The dashed arrows show the vertical distance between the levels

Hence, six thermo-hygrometers (hereafter called RHT probes), manufactured by Rotronic (model HygroLog), were installed in the repository (Fig. 3) to measure T and RH at local time. The uncertainties of the Pt100 resistance thermometers for T and thin film capacitive sensors for RH, respectively equal to ± 0.3 °C and ± 0.8% (from 10 to 60% at 23 °C), were in accordance with the current European Standards on the instruments recommended in cultural heritage conservation [26, 27]. The RHT probes were positioned on the shelves at about 1.8 m above the floor. RHT probes numbered from 1 to 4 were deployed in the area preserving the Ancient collections on each of the three levels of the repository, while RHT5 and RHT6 were installed in the area preserving the Modern collection on the middle level of the repository along the major axis. The sampling frequency was set to 15 min to catch short-term fluctuations due to consultation and management by the staff.

A HOBO U12-012 datalogger was used on a single week from July 27th to August 3rd 2020 to measure the illuminance near the shelves opposite the east-facing windows (Fig. 3).

The outdoor T and RH data used in this analysis were provided by a meteorological station installed on the roof of Fermi building of the Physics department (27.6 m above ground) within the Sapienza University Campus. This ground-based meteorological station (Vaisala Weather Transmitter WXT520), managed by the OMD Foundation (Fondazione Osservatorio Milano Duomo), belongs to the national private network of certified quality urban weather stations “Climate Network” [28].

### Indoor climate characterisation

The quality of the long time series of microclimate data collected in the Alessandrina repository was assessed through the Completeness Index (CoI) defined in [29] before performing data analysis.

Spatial gradients were calculated to evaluate whether relevant differences existed among the hygrothermal conditions detected in the various monitoring positions of the repository. The vertical gradient between the air temperatures measured at RHT3 (middle level) and RHT4 (upper level) was rescaled with respect to the distance of 10 m to evaluate the indoor air stability by comparing it to the dry adiabatic gradient of vertical temperature of − 0.1 °C/10 m. If the vertical temperature gradient (ΔT/Δz) is higher than dry adiabatic gradient of vertical temperature, the air parcel tends to rise (unstable condition) and vice versa.

The moving averages of T and RH were calculated over sliding time windows consistent with the typical response time of paper books to T and RH changes, i.e., 24 h for T and 30 days for RH [15]. The short-term fluctuations of T (i.e., ΔT_24h_) were evaluated as the differences between the T readings and the centred 24-h T moving average (T_24h_). The short-term fluctuations of RH (i.e., ΔRH_30d_) were evaluated as the differences between the RH readings and the centred 30-day RH moving average (RH_30d_).

### Conservation risk assessment

#### Mechanical risk

The European standard EN 15757:2010 [12] was used to reconstruct the historical climate in the Alessandrina repository, i.e., the climatic conditions to which organic hygroscopic collections have become acclimatised. The bands of tolerable RH variations of the historical climate were obtained superimposing on RH_30d_ (i.e., the seasonal RH levels) the lower and the upper limits, calculated respectively as the 7th and the 93rd percentiles of the distribution of the short-term fluctuations ΔRH_30d_. When ΔRH_30d_ departs by less than 10% from the seasonal RH levels, these limits can be considered unnecessarily strict and thus the RH band can be calculated as the RH_30d_ ± 10%.

#### Chemical risk

The validated dose–response function for paper derived by Strlič et al. [13] was used to model the rate of paper degradation per year as a function of pH and the indoor T and RH values. It has to be stressed that pH of paper in the formula is an operationally defined parameter for paper acidity and was not measured on a true solution, as reported in [30] and was not the acidity of a true solution. Then, based on Ekenstam’s equation, the expected lifetime (EL) of paper (i.e., the time in years required for objects to become unfit for use) was derived as a function of the initial DP (DP0) and the critical DP at which objects are no longer suitable for handling [13]. It is worth considering that, since the definition of the EL does not consider natural and artificial light, the results might underestimate the chemical risk to the collections in the Alessandrina repository.

The recently proposed Time Weighted Expected Lifetime (TWEL) index was used to estimate the expected lifetime of paper collections on both a yearly and a seasonal basis [9]. As the well-established Time Weighted Preservation Index (TWPI) defined in [9], the TWEL index integrates the concept of a time weighted index to quantitatively compare the chemical risk due to the indoor climate conditions over different time windows (e.g., yearly or seasonally). The TWEL was defined as:

1 $${\text{TWEL = }}\frac{{\text{n}}}{{\sum\nolimits_{{\text{i = 1}}}^{{\text{t}}} {{\text{EL}}_{{\text{i}}}^{{ - 1}} } }}$$

where n is the total number of observations in the selected time window and the denominator is the cumulative sum of the i-th reciprocals of EL. The detailed description of the procedure followed to calculate the TWEL is provided in [9]. Since the calculation of the TWEL was purposely based on T_24h_ and RH_30d_ (corresponding to the typical response time of paper books), it takes into account the time needed for paper books to reach the equilibrium with the hygrothermal conditions measured in the environment. Moreover, as the TWEL calculation is based on the reciprocals of EL, its value is clearly more influenced by lower values of EL, thus considering the risk underlying the worst-case scenario.

#### Biological risk

The risk of mould growth was assessed by comparing T and RH observations to the Sedlbauer diagram with the curves describing the times for spore germination and mycelial growth (known as isopleths) of *Aspergillus versicolor* [19]. As recently proposed in [31], the critical RH is the one associated with the Lowest Isopleth for Mould (LIM), i.e., the lowest curve where mould activity is assumed to cease, for an optimal substrate [20], thus assuming a worst-case scenario.

The risk of insect infestation is relatively less studied in terms of dose–response functions [31]. For this reason, the threat of insect pest infestation was evaluated through an index defined in [32] to quantitatively estimate the number of eggs laid (e) by webbing cloth moths (*Tineola bisselliella*) as a function of T:

2 $${\text{e = int}}\left\{ {{\text{130*exp}}\left( {{ - }\left( {\frac{{\frac{{{\text{T}}^{{2}} }}{{{30}}}{ - 30}}}{{{12}}}} \right)^{{2}} } \right)} \right\}$$

Webbing cloth moths very rarely damage books and papers, but a few species of moth can attack and cause damage to textiles (such as those used for book covers) and larvae may occasionally tunnel into leather book bindings [21].

Although no recent fungal colonisation or insect proliferation has been reported by the library staff in the Alessandrina repository, this evaluation was also performed to provide a holistic overview on the deterioration risks potentially induced by the climate.

#### Photodeterioration

In addition to the climate-induced risk assessment based on dose–response and damage functions, colorimetric measurements were performed to objectively assess colour changes [33] and evaluate the possible impact of solar radiation in the east-facing side of the repository.

A digital portable spectrophotometer Konica Minolta (model CM-2600d) was used to obtain the reflectance spectrum and the CIE 2000 colorimetric parameters [34]. This instrument is based on the physical measurement of reflected light, through an integrating sphere, at specific wavelengths (400–700 nm at 10 nm steps) corresponding to the visible light spectrum. The CIE coordinates on the L*a*b* (CIELAB) colour space—i.e., the luminance L*, on a scale from 0 (black) to 100 (white), the chromatic dimension a*, on a scale from + 60 (red) to − 60 (green) and the chromatic dimension b*, on a scale from + 60 (yellow) to − 60 (blue)—were used to derive the colorimetric difference ΔE*, calculated as the Euclidean distance between the points in the CIELAB space [34]. The temporal change time of ΔE* was finally fitted using the exponential curve given in [33]:

3 $${\Delta \text{E}}^{*} { = }\frac{{\text{E}_{\infty } \cdot \text{t}}}{{\text{t}_{\text{s}} + \text{t}}}$$

where E_∞_ is the fitted value of ΔE* at infinite time and t_s_ is the time corresponding to E_∞_/2.

A field campaign following a 4-month schedule was carried out from October 2019 until October 2020 to monitor the cumulative colorimetric changes induced by light on the covers of five already discoloured books exposed to solar radiation. The five books monitored were selected to estimate the residual rate of discolouration affecting the covers that were already photodeteriorated. In this sense, colour changes were used to check whether photodeterioration was still possible on these specimens rather than to quantify the risk for the collection. After calibration with the white standard provided by the manufacturer (white calibration plate Minolta CM-A145), measurements on the book covers were repeated 3 times in 3 different sampling points for each sample and then averaged.

Colorimetric measurements were also performed on an unproofed green target sample of cardboard (made in pure Elemental Chlorine Free cellulose, grammage 200 g/m^2^) to estimate the effect of the luminous exposure in the same position as the discoloured books. In order to compare the colorimetric measurements taken at different times on the green target, the same sampling area and calibration standard were used over the monitoring.

#### Risk Index

The Risk Index (RI), defined in [35] as the percentage of time for which RH and T values are out of the safe ranges for selected risks, was adjusted to the case of the library repository by including the specific climate-induced risks affecting paper collections and used to synthetically express the overall deterioration risks on a yearly basis. The mechanical RI was estimated as the percentage of time in which RH observations are beyond the tolerable RH band of the historical climate. It is worth noticing that, according to the procedure suggested by EN 15757:2010 [12], this value will never exceed 14%. The chemical RI was computed as the percentage of time in which the microclimate conditions are responsible for values of expected lifetime lower than 500 years [13]. The insect proliferation RI was calculated as the total number of eggs laid monthly by the webbing cloth moths over a year compared to the maximum number of eggs that can be theoretically laid in a year (i.e., 1560 eggs, corresponding to 130 eggs per months). The fungal colonisation risk was expressed as the yearly percentage of time in which hygrothermal conditions are favourable to mould germination based on the Sedlbauer isopleths for an optimal substrate [20].

## Results and discussion

### Indoor climate characterisation

The time series collected over the period from August 1st, 2019, to July 31st, 2020 was found to be the most complete (CoI = 1) and therefore used in the following analysis.

**Fig. 4:**
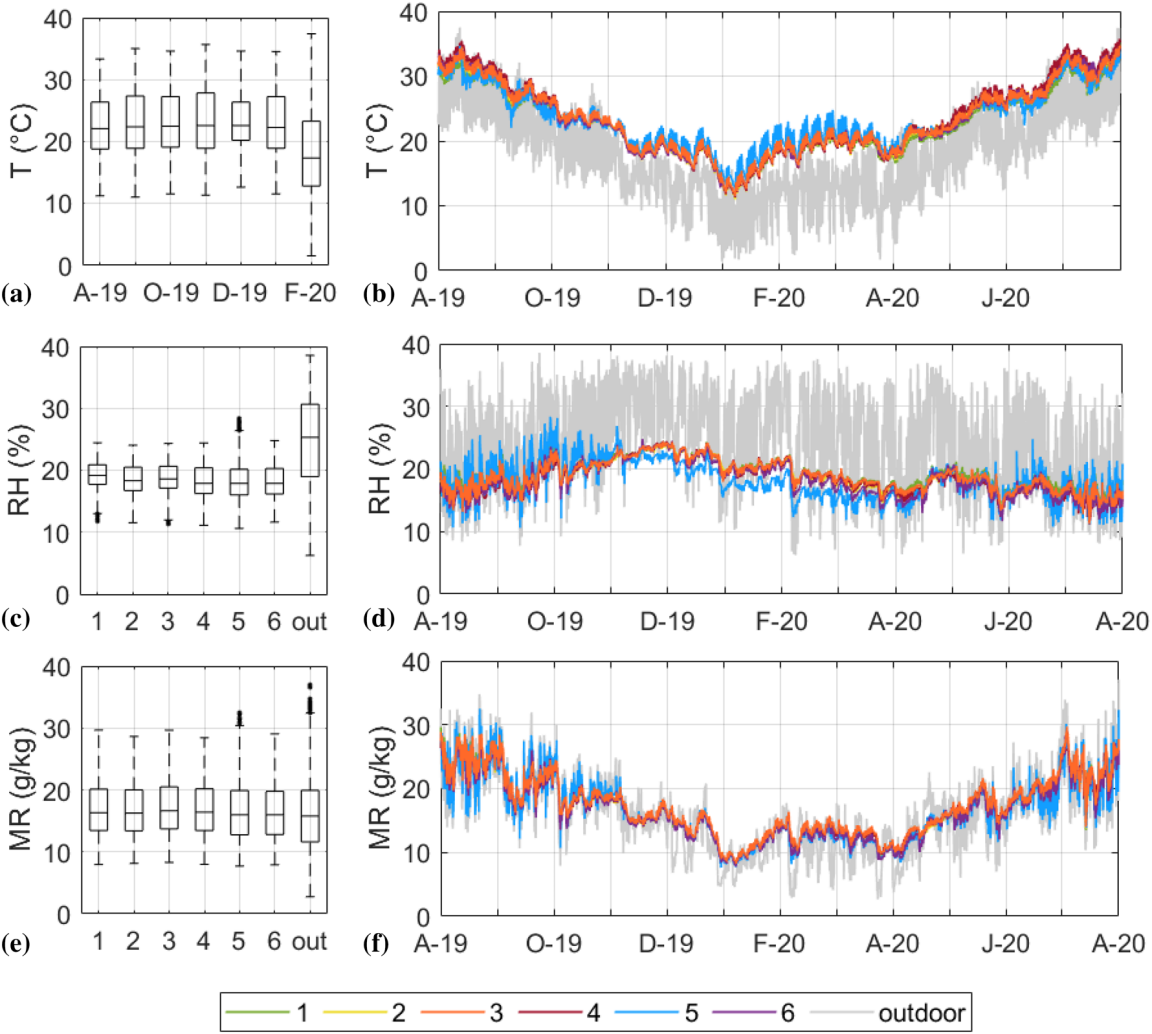
Box-and-whisker plots of indoor (1–6) and outdoor (out) temperature (**a**), relative humidity (**c**) and mixing ratio (**e**) data collected by the six RHT probes and time plots of indoor T (**b**), RH (**d**) and MR (**f**) data. In the box-and-whisker plots, the medians are indicated with horizontal lines dividing each box and the whiskers are set to the lowest and the highest value when they are not outliers (i.e., the values above or below 1.5 × IQR, indicated as black dots)

On a yearly basis, the medians of the indoor T measured by the probes were higher than the outdoor ones (Fig. 4a) and showed comparable levels and trends. Nonetheless, a higher short-term variability can be noticed for RHT5 when looking at the time plot (Fig. 4b) due to the closer proximity to the entrance door, the fan coils, and the west-facing windows (frequently open during the warm season). The indoor RH observations were found to be lower than the outdoor ones (Fig. 4c), with medians of indoor relative humidity between 40 and 50%, as observed in other libraries [9]. The outdoor RH variability, ranging from 20 to 100%, was markedly smoothed out inside the repository (Fig. 4d). The relatively higher variability of the RH observations collected by RHT5 during the warm months (Fig. 4c, d) was probably due to outdoor air entering from the open west-facing windows causing an increased indoor MR variability (Fig. 4e, f).

**Fig. 5:**
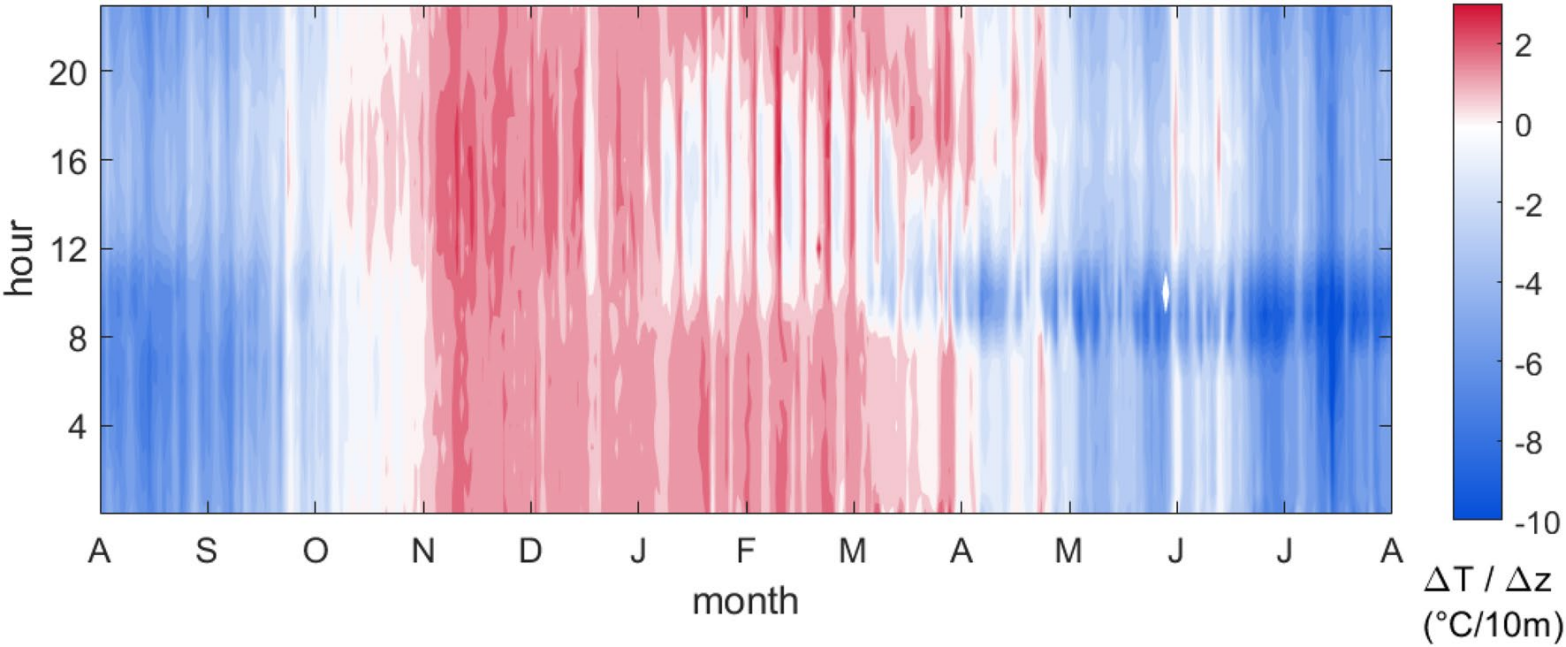
Hourly distribution of vertical temperature gradients (ΔT/Δz, where ΔT = − (T4-T3) compared with the dry adiabatic gradient of temperature of − 0.1 °C/10 m: unstable (red), neutral (white) and stable (blue) conditions

The analysis of the vertical temperature gradients (ΔT/Δz) highlighted that unstable conditions occurred in winter (red areas in Fig. 5), mainly driven by cooler temperatures observed near the ceiling (T4) due to the poorly insulated roof causing heat losses. This effect was partly compensated by the combined effect of heating systems and solar exposure from January to March during weekdays (approximately from 10 a.m. to 7 p.m., visible as white-blue areas in Fig. 5), but positive temperature gradients occurred during weekends, when the heating systems were turned down (red areas in Fig. 5). Unstable conditions determine that air gains buoyancy and forms an upward current, thus tending to increase soiling and transport of dust and fungal spores. As expected, the air temperatures near the ceiling (blue areas in Fig. 5) were higher during the warm season, likely due to the combined effect of increased outdoor temperature caused by the strong radiative exchange and indoor heat accumulation. This condition, being responsible for accelerated rates of chemical deterioration, might affect the conservation of the collections on the upper level. It might be useful to note that the temperature gradient related to stable conditions was steeper than the one related to unstable conditions.

The differences among T and RH data collected by RHT2, RHT3 and RHT6 (deployed on the middle level of the repository) were found to be lower than the instrumental uncertainties. Therefore, we focussed the following analysis on the comparison between the observations collected by RHT3 and RHT5, which were taken as representative respectively of the average conditions surrounding the Ancient collection and the central area of the repository housing the Modern collection.

**Fig. 6:**
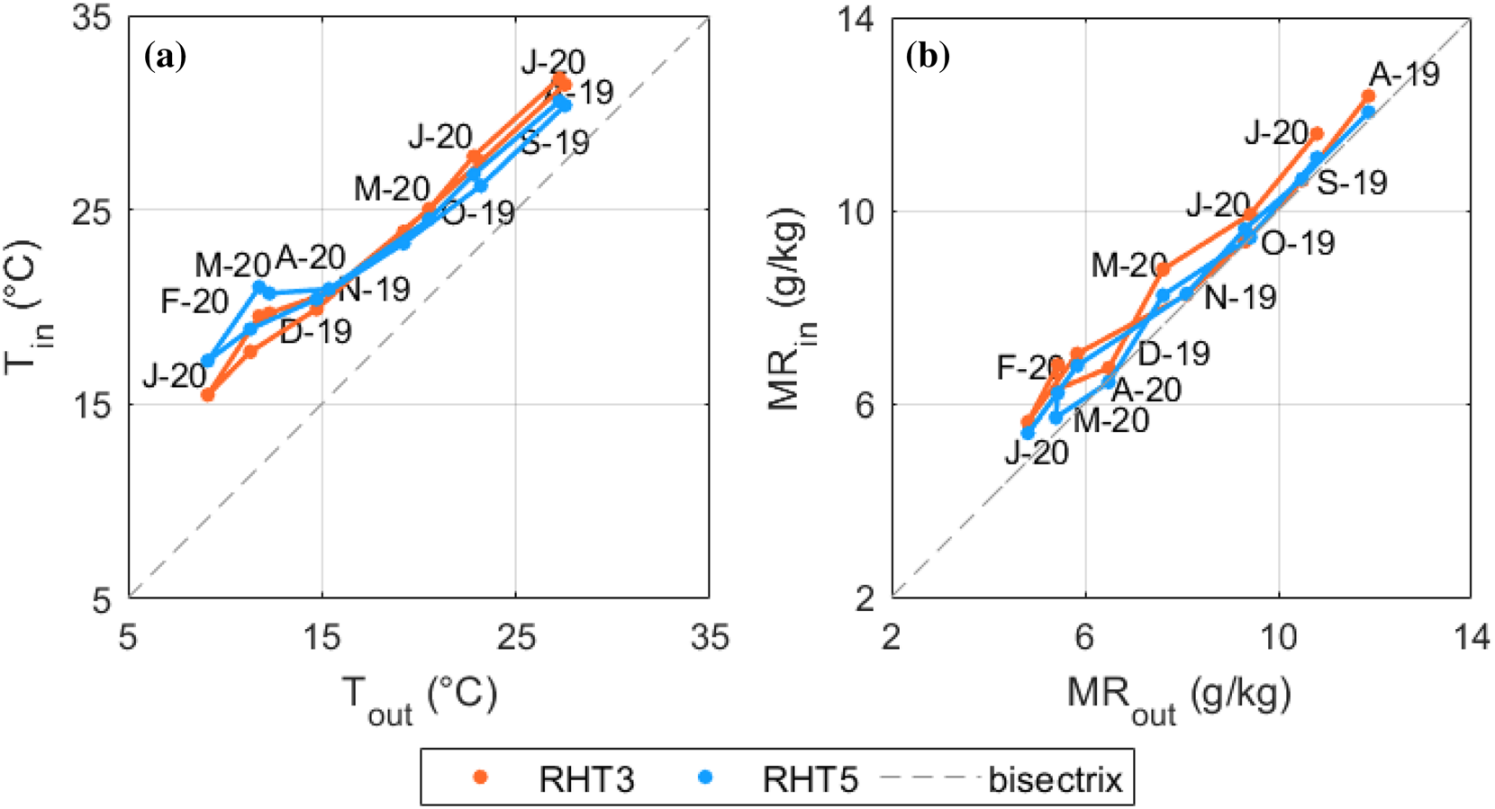
Indoor versus outdoor monthly averages of air temperature (**a**) and mixing ratio (**b**) values

The T and MR data collected by RHT3 and RHT5 were used to investigate the influence of the external climate on the indoor hygrothermal conditions. Looking at the scatter plots reported in Fig. 6, the slopes of the solid lines relating indoor and outdoor T and RH monthly averages at the two sites of the repository were similar. It can be noticed that indoor T values were higher than the outdoor ones, while MR values were mostly identical. If compared to the average T conditions collected by RHT3, the monthly T averages measured by RHT5, in the central area of the repository, were higher in winter (due to the proximity of the fan coils) and slightly lower in summer (being less affected by solar radiation).

**Fig. 7:**
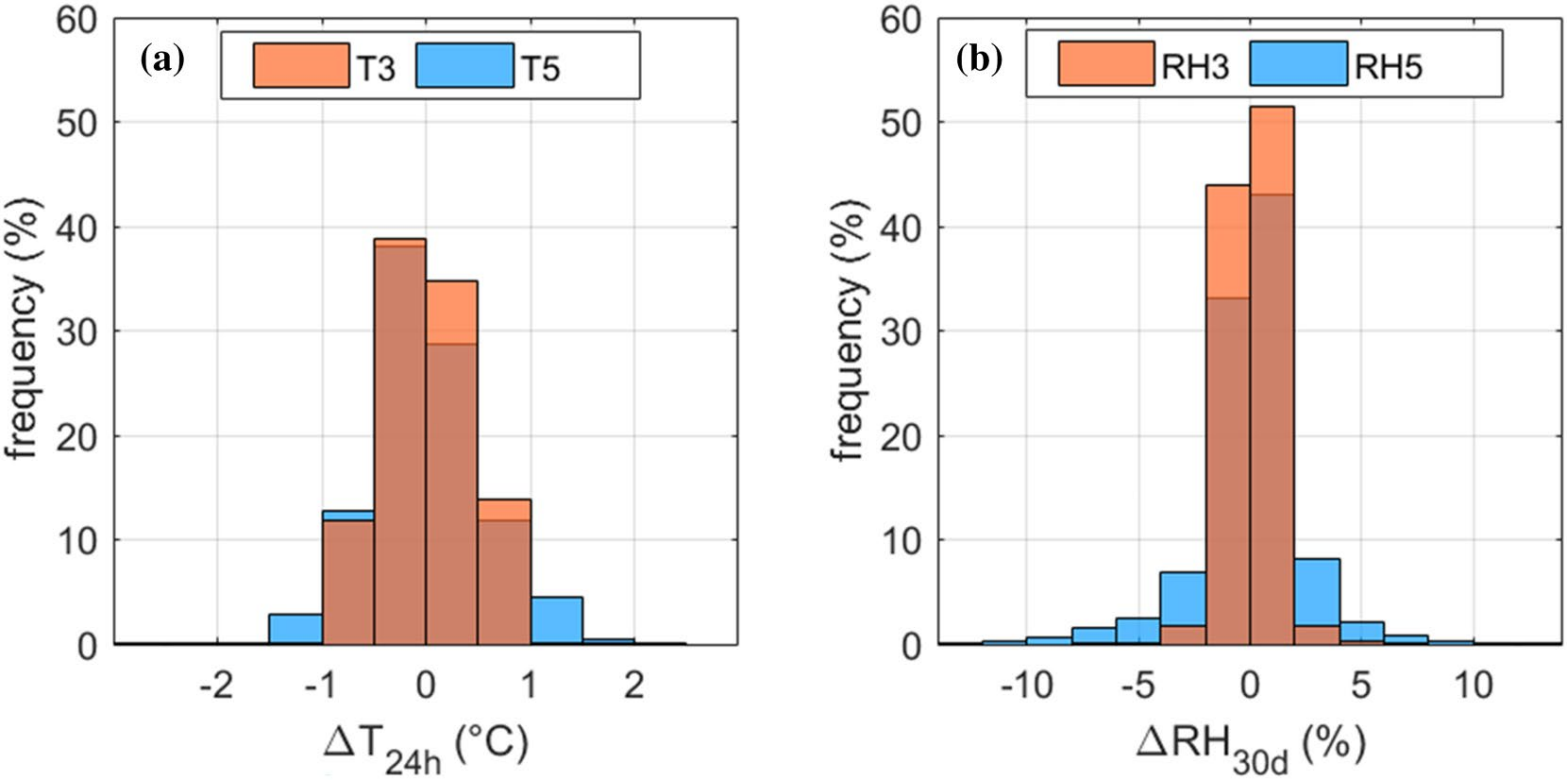
Frequency distribution of differences between T observations and the running average over 24 h (**a**) and between RH observations and the running average over 30 days for RH (**b**)

Figure 7 shows the frequency distribution plots of the short-term fluctuations ΔT_24h_ and ΔRH_30d_. Both ΔT_24h_ and ΔRH_30d_ showed high stability, symmetrically spreading around the zero by about ± 1 °C for T and ± 5% for RH in the case of data collected by RHT3 (Fig. 7a) and by about ± 2 °C for T and ± 10% for RH in the case of data collected by RHT5 (Fig. 7b). Since the sliding time windows used to calculate the moving averages ΔT_24h_ and ΔRH_30d_ were set to be compatible with the typical response time of paper books to T and RH changes [15], these results highlighted that the collections in densely-packed shelved were likely exposed to a limited thermo-hygrometric variability.

### Conservation risk assessment

The observations collected by RHT3 and RHT5 were used to estimate the principal conservation risks for the Ancient collection and the Modern collection, respectively.

**Fig. 8:**
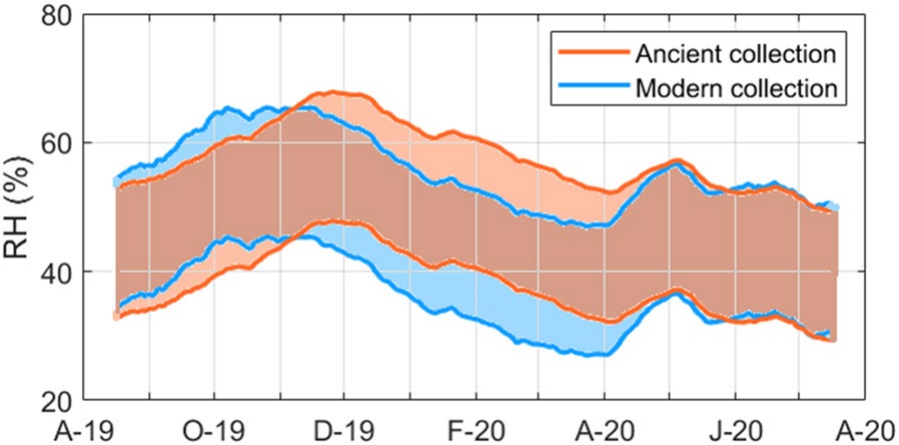
Tolerance RH bands based on the historical climate in the repository. The two 15-day periods at the beginning and the end of the year were cut due to calculation of RH_30d_

The mechanical risk was evaluated reconstructing the historical climate according to EN 15757:2010 [10]. Since RH short-term fluctuations did not exceed the tolerance bands of ± 10% from RH_30d_, the upper and lower limits for Ancient and Modern collections were drawn as RH_30d_ ± 10%. The peaks of the two tolerance RH bands in Fig. 8 were slightly shifted by a time lag of one month; this behaviour could be due to differences in the heating systems and air exchanges in the two repository areas. Since May 2020, they fully overlapped, probably due to a reduced windows’ opening associated with the limited presence of staff during the COVID-19 pandemic situation.

The information on the historical climate (Fig. 8) can be used in management of the collections. Indeed, it was found that any relocation of moisture-vulnerable objects (e.g., composite objects, parchment-bound books) within the repository area can be considered safe from the physical damage induced by strain–stress cycles [10]. Moreover, to avoid tensile stresses in tightly and/or layered bound objects [11] when these items need to be moved to spaces characterised by different hygrothermal condition (as in the case of loan and consultation), the definition of the target tolerance band of RH fluctuations in the new environment must consider the historical climate to which they have acclimatised.

The annual TWEL associated with Ancient and Modern collections was calculated based on ΔT_24h_ and ΔRH_30d_ combining different values of pH and DP0 for various paper types (Table 1) to explore the effect of the microclimate on the chemical deterioration risk for various paper types.

**Table 1:** TWEL values (in years) in relation with T and RH values surrounding Ancient and Modern collections obtained for various paper types by combining values of acidity (pH) and initial degree of polymerisation (DP0)

TWEL	pH	DP0		
		Low (600)	Fair (1500)	Good (2000)
Ancient collection	Acidic (5)	219	677	1188
	Neutral (7)	351	1083	1902
	Basic (8)	373	1151	2021
Modern collection	Acidic (5)	204	631	1107
	Neutral (7)	327	1009	1772
	Basic (8)	348	1072	1882

On a yearly basis, the exploratory evaluation (Table 1) highlighted that the risk due to cellulose hydrolysis can be relevant for all the paper-based objects having DP0 ≤ 600 (low), with a reduction of the expected lifetime of the acidic ones up to approximately 200 years. On the contrary, for paper-based objects with a DP0 from fair (1500) to good (2000), the chemical risk in both the sites was found to be compatible with the 500-year planning horizon typical of conservation strategies for historic libraries. Overall, the chemical risk associated with the T and RH values surrounding the Modern collection was slightly higher for all the explored paper types.

**Fig. 9:**
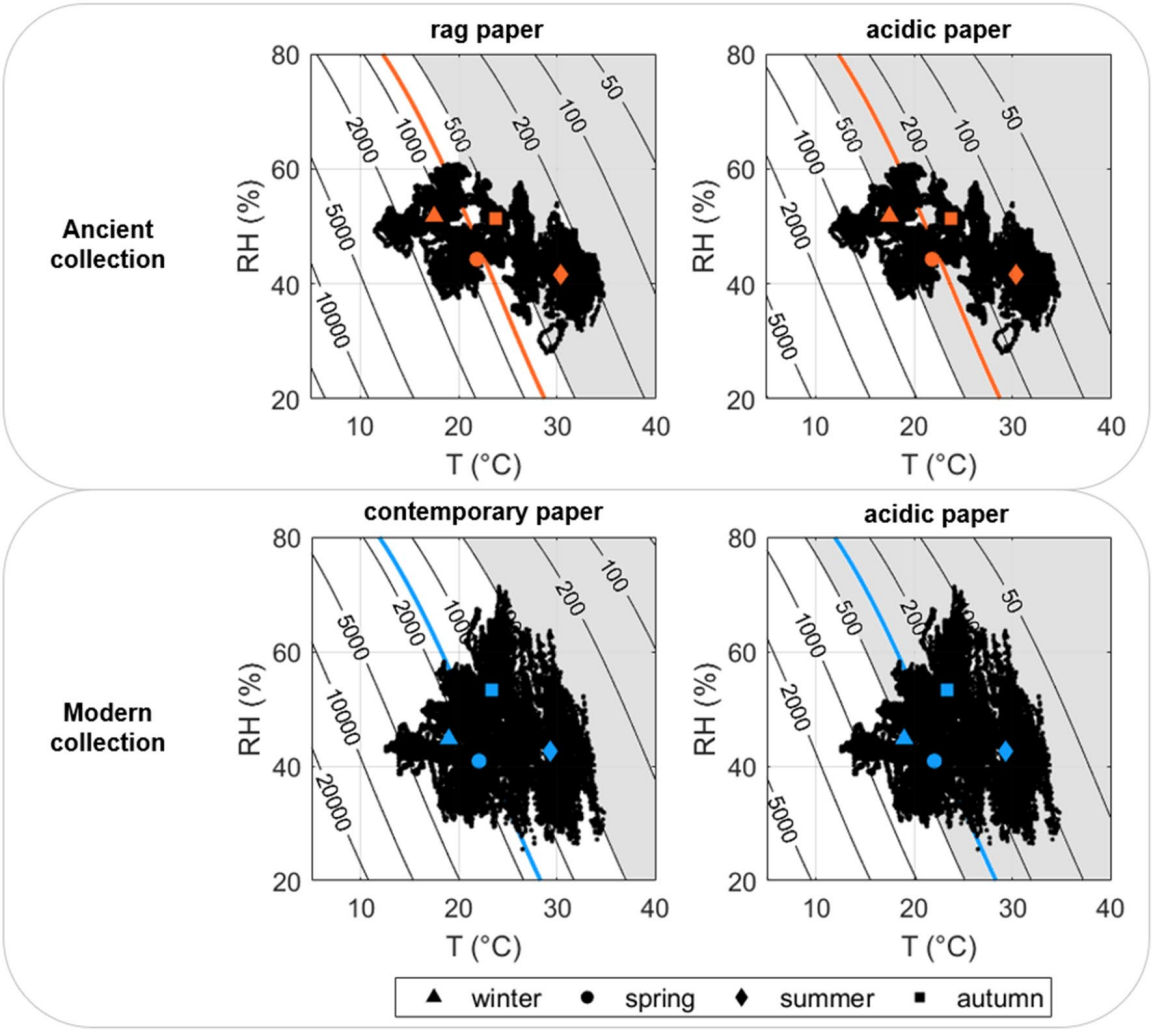
Temperature and relative humidity observations (black dots) plotted on the isochrones of expected lifetime, together with the yearly (coloured curves) and seasonal (coloured markers) TWEL values (in years). Shaded area: T and RH conditions favourable to chemical decay

To further investigate the riskiest period of the year in terms of chemical deterioration, the T and RH observations at the two sites of the repository were compared to the isochrones for acidic, rag and contemporary paper types (Fig. 9). A prevalence of rag paper in the Ancient collection and of contemporary paper in the Modern collection was assumed based on the production period of the books. The chemical risk for the acidic items in the collections was also evaluated, as they are the most vulnerable to cellulose hydrolysis. The seasonal TWEL (coloured markers in Fig. 9) highlighted that, although the annual values (coloured curves in Fig. 9) exceeded the 500-year planning horizon for conservation [13], the T and RH conditions in summer and autumn were favourable to the chemical decay of rag paper in the Ancient collection. In contrast, acidic paper can be subject to cellulose hydrolysis during most of the year, with winter as the only exception. Contemporary paper is at no risk on a seasonal basis, but the high temperatures (i.e., T > 25 °C) occurring in summer could threaten its durability, particularly when associated with high relative humidity values (i.e., RH > 60%).

**Fig. 10:**
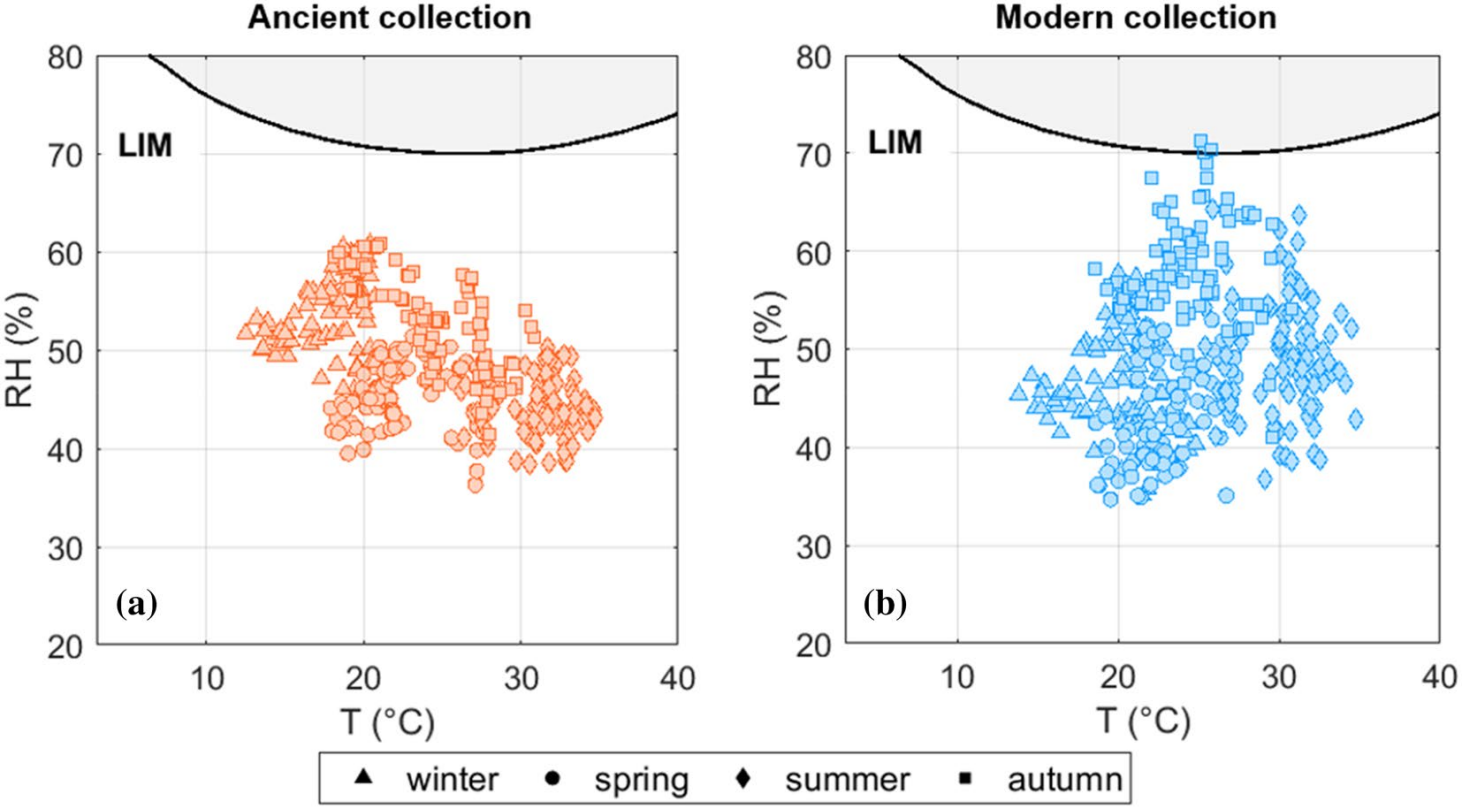
Lowest isopleths for spore germination and mycelium growth (LIM) for optimal substrate according to Sedlbauer [20] and maximum daily T and RH values. Shaded area: RH conditions exceeding the threshold favourable to fungal decay

In Fig. 10 the maximum daily T and RH values experienced by Ancient (Fig. 10a) and Modern (Fig. 10b) collections were compared to the lowest isopleths for spore germination and mycelium growth for optimal substrate [20]. The results highlighted that on few cases during autumn the risk of fungal colonisation was possible for the Modern collection, should conditions of low ventilation and water vapour condensation occur on cold metal surfaces (such as those of the repository shelves).

**Fig. 11:**
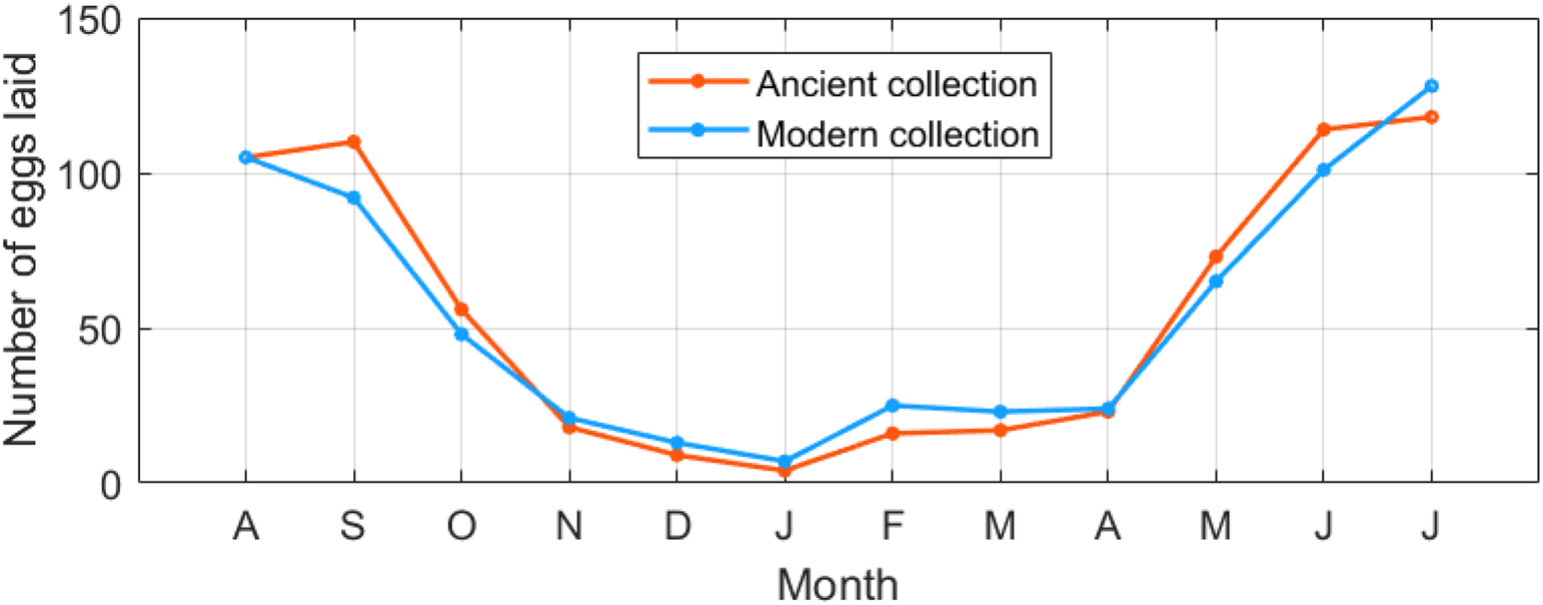
Number of eggs laid by the webbing cloth moth (*Tineola bisselliella*) as a function of the average monthly temperature values in the repository

The risk of insect proliferation was estimated in Fig. 11 as a function of the monthly T average values in the repository. The expected number of eggs laid by the webbing cloth moth (*Tineola bisselliella*) was found to be practically equal for Ancient and Modern collections. During the monitored year, it was higher than 50 from May to October, with a slight inversion of the trend in August due to T > 30 °C; in January, the expected amount of moth eggs decreased up to the minimum value of 4 eggs due to T means below 20 °C.

The illuminance measured in the proximity of the book covers in August 2020 during the morning hours exceeded 32,000 lx (i.e., the upper measurement limit of the light sensor). The total luminous exposure measured within a single week, higher than 5.2 Mlx, was approximately ten times higher than the maximum annual luminous exposure recommended by CEN/TS 16163:2014 [36] for low sensitivity materials (i.e., 0.6 Mlx ˑh per year).

Table 2 shows the average values and variability of the total colorimetric difference (ΔE*) measured on 5 book covers over the field campaign carried out from October 2019 to October 2020. ΔE* was found to be always high (ΔE* > 30). Indeed, since the selected samples were already visibly photodeteriorated, the measured colorimetric difference considerably exceeded ΔE* = 6, i.e., the perceptible and non-acceptable threshold of colorimetric difference from the original colour [37]. ΔE* measurements did not change significantly over 1 year (half maximum spread among ΔE* always lower than 7), meaning that the discolouration rate was already too low to be observed in such a short time window.

**Table 2:** Mean and half maximum spread of the colour changes (ΔE*) measured on 5 book covers (BC) samples from October 2019 to October 2020 following a 4-month schedule

Sample	ΔE*	
	Mean	Half maximum spread
BC1	128	2
BC2	32	4
BC3	34	7
BC4	94	1
BC5	78	4

**Fig. 12:**
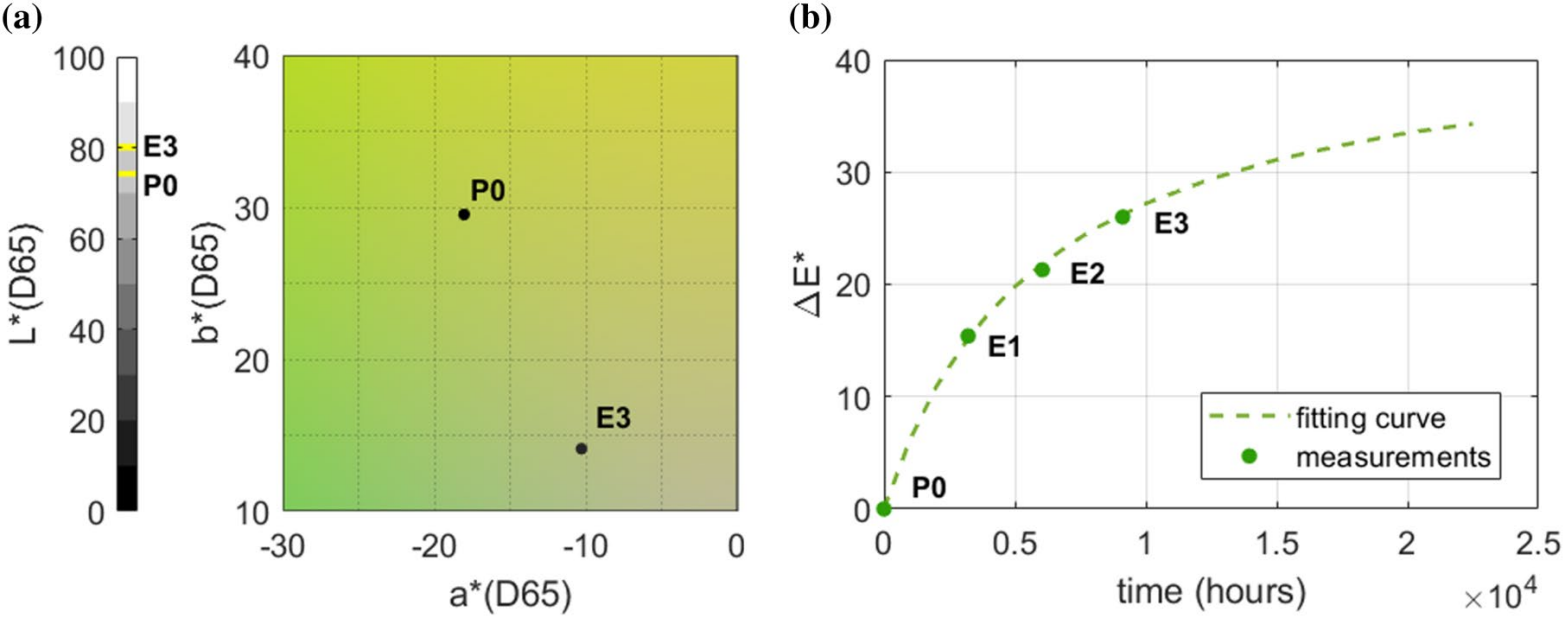
Colorimetric coordinates of the points measured on the green mock sample (**a**) and total colorimetric difference ΔE* as a function of time (**b**). The measured data (dots) were used to fit the curve in Eq. 3 (green dashed line) given. P0 = protected area (October 2019) and E1, E2, E3 = areas exposed to light (February, June and October 2020, respectively)

The evolution of the colorimetric change measured from October 2019 to October 2020 on the target sample of green cardboard are shown in Fig. 12. The colorimetric change observed on the target sample followed a typical exponential trend, with the slope of ΔE* rapidly changing from the initial conditions (P0) to those measured after the first 4 months of luminous exposure (E1) and then slowly decreasing in the following spring and summer (E2 and E3), even if these seasons are typically associated with a higher dose of solar radiation than winter.

**Fig. 13:**
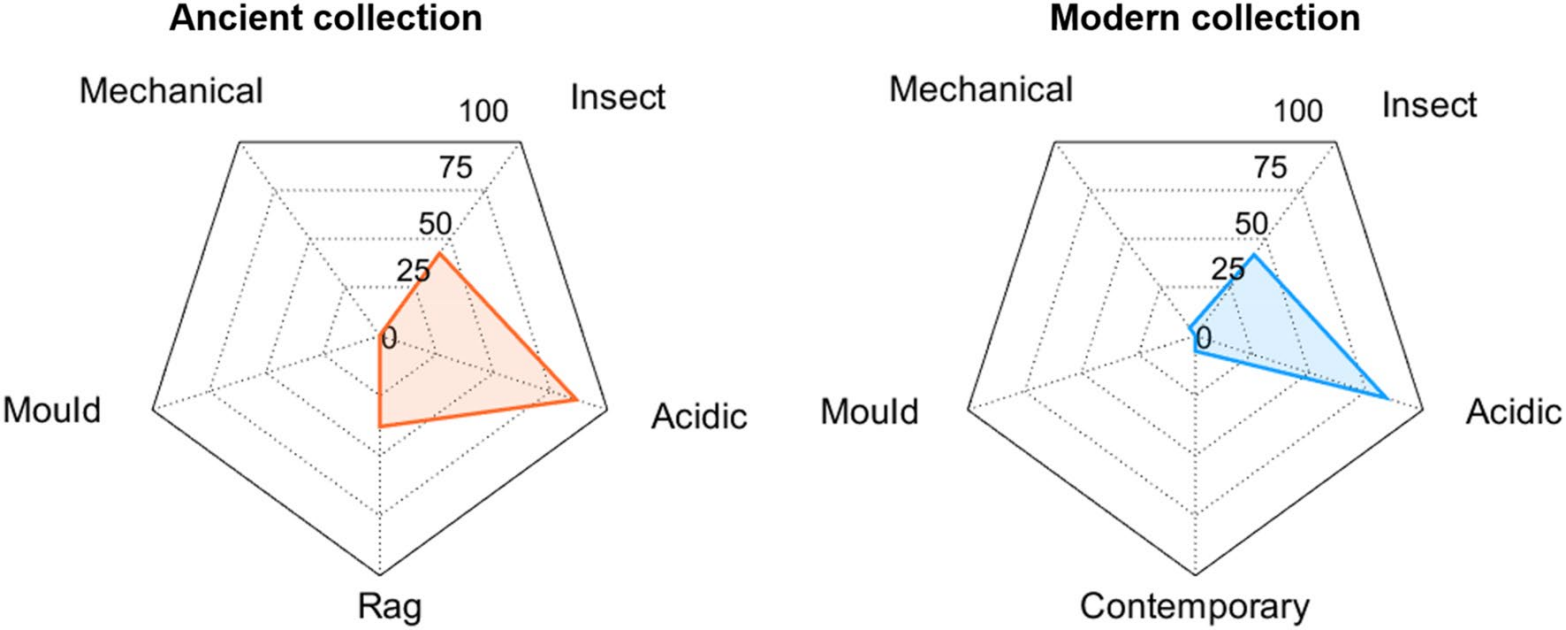
Radar plot of the Risk Index (%) calculated on a yearly basis for the mechanical [12], chemical [9] and biological risks [20, 32]

Figure 13 shows the radar plots with the Risk Index to compare the climate-induced risk for the Ancient and Modern collections. The coloured area indicates the magnitude (in percentage of time) of the total mechanical, chemical and biological risk over a year. The risk of cellulose hydrolysis for acidic paper associated with the hygrothermal conditions surrounding the Ancient and Modern collections was consistently high (RI = 86% and 84%, respectively), while for less vulnerable paper types it was equal to RI = 38% for rag paper in the Ancient collection and to RI = 6% for contemporary paper in the Modern collection. Insect proliferation in the repository was favoured during approximately 42% of the year. In contrast, the risk of mechanical deterioration and mould germination was negligible for both collections.

## Conclusions

A global characterisation of the main climate-induced deterioration risks for paper collections (mechanical stress, cellulose hydrolysis, mould and insect proliferation) was carried out in the repository of the Alessandrina Library (Rome, Italy).

Temperature (T) was found to be the key microclimate stressor to be monitored for preventive conservation of paper collections, as it controlled the rate of cellulose hydrolysis while favouring insect proliferation. The Time Weighted Expected Lifetime (TWEL) highlighted that acidic paper could be subject to cellulose hydrolysis during most of the year, while rag paper only in summer and autumn; on the contrary, contemporary paper was mostly at no risk. The study of the vertical T gradients highlighted that unstable conditions occurred in winter, likely increasing soiling and transport of dust and fungal spores; on the contrary, heat accumulated on the upper levels of the repository during the rest of the year, causing accelerated chemical rates. Relative humidity (RH) in the repository was below 60% for most of the year, although fungal colonisation might occur during autumn in poorly ventilated microenvironments (e.g., water vapour condensation on cold surfaces of the metal shelves). The tolerable RH conditions were defined based on the “historical climate” to reduce mechanical deterioration risk of moisture-vulnerable objects in the event of loans, relocation, and consultation. Moreover, since the estimated luminous exposure in the repository was found to be incompatible with conservation of materials sensitive to photodeterioration, any deployment of unaltered photosensitive materials on the shelves near the windows would be risky, unless protections from direct exposure to solar radiation were to be installed. A limitation of the study was the lack of appropriate instruments for the measurement of air exchange rate in the repository. This information would have been useful to study the effect of windows opening on the indoor environment in terms of heat/moisture exchanges with the outdoors.

The study highlighted that high temperatures and solar exposure are responsible for the major deterioration risks affecting the collections. For this reason, two passive retrofit measures could be suggested: 1) improving roof insulation to mitigate summer temperature peaks and winter vertical gradients causing unstable conditions; 2) adopting shading devices (e.g., blinds, UV-filter films on the window panes) to reduce solar radiation entering from the large east-facing windows. The benefit of the retrofit interventions could be preliminarily evaluated through whole-building dynamic simulation [38, 39]. A further measurement campaign could be planned during and after the retrofit to quantify its effectiveness on both the indoor intensity of radiation and air temperature.

Although the research focussed on a specific case study, a similar approach could be effectively adapted to most library and archival repositories conserving paper-based collections with different environments and conservation needs. For this reason, future developments will design a standalone executable of the Risk Index algorithm to better support directors of libraries and other stakeholders for refining and automatising the risk assessment procedure.

## Data Availability

The datasets used and/or analysed during the current study are available from the corresponding author on reasonable request.

## Abbreviations

BC: Book cover
CoI: Completeness Index (-)
CIE: Commission Internationale de l’Eclairage
D65: Standard illuminant for the average Daylight in Western Europe correlated to a colour temperature of approximately 6500 K
e: Number of eggs laid by webbing cloth moths (-)
EL: Expected Lifetime (-)
HVAC: Heating, Ventilation, and Air Conditioning
IQR: Interquartile range
LIM: Lowest Isopleth for Mould
RHT: Probes measuring T and RH
TWEL: Time Weighted Expected Lifetime (years)
TWPI: Time Weighted Preservation Index (-)

## Symbols

Δa*: Colorimetric difference in red and green (-)
Δb*: Colorimetric difference in yellow and blue (-)
ΔE*: Total colorimetric difference (-)
ΔL*: Colorimetric difference in lightness and darkness (-)
ΔRH_30d_: Difference between T reading and RH_30d_ (%)
ΔT: Vertical temperature gradient (°C)
ΔT_24h_: Difference between T reading and T_24h_ (°C)
Δz: Vertical distance between two probes (m)
DP: Paper degree of polymerisation (-)
DP0: Initial DP (-)
E_∞_: Fitted value of ΔE* as time approaches infinity (-)
MR: Mixing Ratio (g∙kg^−^^1^)
pH: Parameter for acidity of paper (-)
RH: Relative humidity (%)
RH_30d_: Centred 30-day moving average of relative humidity (%)
t: Time (hours)
T: Air temperature (°C)
t_s_: Time corresponding to E_∞_/2 (hours)
T_24h_: Centred 24-h moving average of temperature (°C)
